# Mapping distribution of brain metastases: does the primary tumor matter?

**DOI:** 10.1007/s11060-020-03419-6

**Published:** 2020-02-17

**Authors:** T. Schroeder, P. Bittrich, J. F. Kuhne, C. Noebel, H. Leischner, J. Fiehler, J. Schroeder, G. Schoen, S. Gellißen

**Affiliations:** 1 Department of Diagnostic and Interventional Neuroradiology, University Medical Center Hamburg-Eppendorf, Gebaeude O22, Martinistr. 52, 20246 Hamburg, Germany; 2 Department of Radiology, Schoen Klinik Hamburg Eilbek, Hamburg, Germany; 3 Department of Neurology, University Medical Center Hamburg-Eppendorf, Hamburg, Germany; 4 Department of Medical Biometry and Epidemiology, University Medical Center Hamburg-Eppendorf, Hamburg, Germany

**Keywords:** Magnetic resonance imaging, Brain metastasis, Brain metastases, Primary tumor type, Distribution

## Abstract

**Purpose:**

Prior reports on the location and sizes of brain metastases almost entirely focus on patients with primary breast and pulmonary cancer. This is the first study comparing multiple other types of cancer that metastasize to the brain.

**Methods:**

This monocentric retrospective study includes 369 untreated patients with 3313 intraaxial brain metastases. Following semi-manual segmentation of metastases on post-contrast T1WI, cumulative spatial probability distribution maps of brain metastases were created for the whole group and for all primary tumors. Furthermore, mixed effects logistic regression model analysis was performed to determine if the primary tumor, patient age, and patient sex influence lesion location.

**Results:**

The cerebellum as location of brain metastases was proportionally overrepresented. Breast and pulmonary cancer caused higher number of brain metastases to what would normally be expected. Multivariate analyses revealed a significant accumulation of brain metastases from skin cancer in a frontal and from breast and gastrointestinal cancer in a cerebellar location.

**Conclusion:**

Distribution of brain metastases is very heterogeneous for the distinct primaries, possibly reflecting the diversity of mechanisms involved in brain metastases formation. In daily clinical practice distribution patters may be beneficial to predict the primary cancer site, if unknown.

**Electronic supplementary material:**

The online version of this article (10.1007/s11060-020-03419-6) contains supplementary material, which is available to authorized users.

## Introduction

Brain metastases (BM) as secondary brain neoplasms are the most common type of brain tumors in adults [1]. Incidence was shown to be 14/100.000 per year, which markedly exceeds the frequency of primary brain tumors (7.25) [2, 3]. Overall, BM occur in 8.5–9.6% of cancer patients following hematogenous spread. The most common primary tumors are pulmonary (39–56%), breast (13–30%), skin (8–11%), gastrointestinal (6–9%), and renal cancer (2–6%) [4–12]. In 2–14% of cases of BM the primary tumor is unknown [9–12].

Prognosis of patients with metastatic disease of the brain was shown to be very poor with an overall survival (OS) of one to 2 months if untreated [13]. OS in BM patients can improve to four to six months if treated with systemic therapies, surgery, or radiation. Only certain forms of non-small cell lung cancer (NSCLC) and human epidermal growth factor receptor 2-positive breast cancer were shown to have a better prognosis [14–16]. A majority of BM patients will eventually be treated with whole brain radiation therapy (WBRT) which has severe short and long term side effects such as fatigue, dermatitis, and neurocognitive impairment [17]. Thus, further elucidation of the underlying principles of BM formation is critical.

Only few studies have examined the lobar distributional pattern of BM and virtually all of them focus on breast and pulmonary cancer whereas there is only one report mapping distribution of other primary tumor types [18–21]. This is the first study that examines the BM dissemination and size of multiple other primary tumor entities that metastasized to the brain resulting in an atlas illustrating brain metastases distribution for different tumor groups at a glance. We hypothesized that there are detectable differences in BM distribution between the groups and that some patterns may be specific for a certain primary site.

## Materials and methods

### Study cohort

This monocentric study includes a total of 369 patients with an age of ≥ 18 years who presented to our university medical center from 2014 to 2016 and were newly diagnosed with at least one intraaxial BM. Primary tumor type was determined according to the pathology report of the BM. If surgery or biopsy of BM was not performed we used histology of extracerebral biopsy as reference. Patients with more than one type of cancer who lacked BM surgery or biopsy were excluded from analysis. Due to the retrospective character of this study, our local ethics committee waived informed consent (Ethik-Kommission der Aerztekammer Hamburg, WF-018/15). Demographic (sex and patient age at diagnosis of primary tumor and at diagnosis of BM) and BM-related data (primary tumor type) were collected.

### MRI study protocol

MRI was performed using a 1.5 Tesla (Magnetom Sonata, Siemens Healthcare, Erlangen, Germany; Magnetom Symphony, Siemens Healthcare, Erlangen, Germany, and Magnetom Avanto, Siemens Healthcare, Erlangen, Germany) in 330 patients or a 3 Tesla scanner (Magnetom Skyra, Siemens Healthcare, Erlangen, Germany; Ingenia, Philips Medical Systems, Best, The Netherlands) in 39 patients.

Axial three-dimensional gradient echo T1WI was performed in 306 patients and T1WI spin echo with flow compensation in 63 patients following weight-adjusted IV Gadolinium injection. Sequence parameters (TR, TE, TI, FOV, matrix, pixel size, slice thickness, interslice gap, and number of slices) varied among the different scanners and were published earlier [22].

### Image analysis

In all patients we detected and semi-manually segmented a total of 3313 BM on T1WI images aided by the Analyze Software System 11.0 (Biomedical Imaging Resource, Mayo Clinic, Rochester, MN, USA) [23]. Segmentation was also performed in one patient who showed two connecting metastases. Here, an experienced neuroradiologist manually adjusted the lesion margins. Afterwards, T1WI images were automatically co-registered to the 1 mm Montréal Neurological Institute standard space using the Oxford Centre for Functional Magnetic Resonance Imaging of the Brain Software Library 5.0 (Analysis Group, Oxford, UK) linear (affine) registration tool. Correct registration of all T1WI and the BM segmentations to the Montréal Neurological Institute space was secured through visual inspection by two readers *(T. S. and S. G.).* Based on anatomical regions defined by the Montréal Neurological Institute atlas cumulative spatial probability distribution maps were created both for the whole cohort and for the primary tumor entities.

### Statistical analysis

Statistical analysis was conducted using IBM SPSS Statistics® software (IBM® 2011, version 20, Armonk, New York, USA). In order to determine if the primary tumor entity, patient age, and patient sex had an influence on the 5 commonest BM locations (see results section), mixed effects logistic regression model analysis was run including the patient identifier as random effect and primary tumor type, decades of age (at the time of diagnosis of BM), and sex as fixed effects. In order to illustrate the results of multivariate analysis we used boxplots showing the frequency of the respective tumor group lobe wise compared to the expected metastatic rate (Fig. 3). The expected metastatic rate (vertical line in Fig. 3) was determined by calculating the respective lobe volume as part of the whole brain volume in the MNI space. Assuming that BM occur with the same frequency throughout the brain then lobe volume would determine the lobar number of BM (or: the BM probability that would statistically be expected in this region).

If not otherwise indicated, data are given as median (interquartile range).

## Results

### Demographics

369 patients fulfilled our inclusion criteria (185 females and 184 males). Women had a median age of 60 (51.5–68) years at diagnosis of the primary tumor and 63 (54–70) years at diagnosis of BM with a latency of 12 (0–39) months between the two events. Men had a median age of 61 (52–70.75) years at diagnosis of the primary tumor and 62 (53.25–72.75) years at diagnosis of BM with a latency of 9 (0–21) months in between.

In our cohort the following primary tumor entities were represented:

- Pulmonary cancer (167 patients: 120 NSCLC, 46 small cell lung cancer, 1 unknown),
- Breast cancer (47 patients),
- Skin cancer (45 patients: 43 melanoma, 1 Merkel cell carcinoma, and 1 squamous cell carcinoma),
- Genitourinary cancer (45 patients: 17 kidney, 10 prostate, 6 urothelial cell, 5 ovarian, 5 testicular, and 2 uterine cancer),
- Gastrointestinal cancer (36 patients: 12 colon, 8 rectal, 7 esophageal, 4 gastroesophageal, 1 gastric, 1 neuroendocrine, 1 duodenal, 1 gallbladder, and 1 cholangiocellular carcinoma),
- Cancer of unknown primary (18 patients),
- Sarcoma (9 patients),
- Head and neck (2 patients: 1 tonsillar and 1 thyroid cancer), see Table 1.

**Table 1:** Distribution of the number of patients, number of BM, volume of single BM, age and gender per primary tumor entity

Primary tumor	N patients/369 (%)	N BM/3313 (%)	N BM per patient	Single BM volume in cc	Age in years	Gender (n male (%)/n female (%) of 369 patients)
All patients	369 (100)	3313 (100)	3 (1–6.5)	0.08 (0.03–0.32)	62 (54–71.5)	184 (49.9)/185 (50.1)
Pulmonary	167 (45.3)	1619 (48.9)	3 (1–6)	0.07 (0.03–0.26)	64 (55–72)	89 (24.1)/78 (21.1)
NSCLC Adenocarcinoma	118 (32.0)	934 (28.2)	3 (1–6)	0.06 (0.02–0.25)	63 (55–72)	59 (16.0)/59 (16.0)
NSCLC Large-cell carcinoma	2 (0.5)	11 (0.3)	5.5 (2–)	0.27 (0.07–1.77)	63 (62–)	0/2 (0.5)
SCLC	46 (12.5)	673 (20.3)	3 (2–14)	0.09 (0.03–0.27)	67.5 (59.25–73.25)	29 (7.9)/17 (4.6)
Unknown	1 (0.3)	1 (0.0)	1	20.6	52	1 (0.3)/0
Breast	47 (12.7)	669 (20.2)	3 (1–11)	0.01 (0.02–0.21)	55 (46–69)	3 (0.8)/44 (11.9)
Invasive ductal	20 (5.4)	315 (9.5)	3.5 (2–9.5)	0.03 (0.01–0.12)	57 (46.25–70.5)	2 (0.5)/18 (4.9)
Invasive lobular	2 (0.5)	18 (0.5)	9 (2–)	0.15 (0.09–0.44)	64 (61–)	0/2 (0.5)
Inflammatory	2 (0.5)	13 (0.4)	6.5 (5–)	0.44 (0.12–4.86)	58.5 (47–)	1 (0.3)/1 (0.3)
NST	11 (3.0)	223 (6.7)	11 (2–36)	0.06 (0.02–0.25)	51 (46–69)	0/11 (3.0)
Unknown	12 (3.3)	1 (0.0)	1 (1–3)	0.10 (0.04–0.49)	57 (41.5–68)	0/12 (3.3)
Skin	45 (12.2)	326 (9.8)	2 (1–5)	0.11 (0.03–0.56)	65 (52–76)	27 (7.3)/18 (4.9)
Melanoma	43 (11.7)	323 (9.7)	2 (1–6)	0.12 (0.03–0.57)	65 (52–74)	26 (7.0)/17 (4.6)
Merkel cell	1 (0.3)	2 (0.1)	2	0.09 (0.02–)	81	0/1 (0.3)
Squamous cell carcinoma	1 (0.3)	1 (0.0)	1	0.01	89	1 (0.3)/0
GU	45 (12.2)	348 (10.5)	3 (1–5)	0.10 (0.03–0.40)	61 (55–73)	26 (7.0)/19 (5.1)
Kidney	17 (4.6)	62 (1.9)	3 (1–5.5)	0.12 (0.05–1.68)	63 (56.5–75)	7 (1.9)/10 (2.7)
Prostate	10 (2.7)	95 (2.9)	3 (2–5.5)	0.02 (0.01–0.05)	65 (60.75–73.75)	10 (2.7)/0
Urothelial cell	6 (1.6)	74 (2.2)	9.5 (2.5–22.75)	0.25 (0.09–0.63)	62 (52–69.25)	4 (1.1)/2 (0.5)
Ovarian	5 (1.4)	9 (0.3)	5 (1–3)	0.78 (0.02–5.53)	59 (53–71)	0/5 (1.4)
Testicular	5 (1.4)	106 (3.2)	5 (1.5–49)	0.13 (0.03–0.27)	35 (27–40.5)	5 (1.4)/0
Uterine	2 (0.5)	2 (0.1)	1	2.3 (2.01–)	51.5 (45–)	0/2 (0.5)
GI	36 (9.8)	175 (5.3)	2 (1–5.5)	0.09 (0.04–0.53)	59 (54.25–67)	20 (5.4)/16 (4.3)
Colon	12 (3.3)	81 (2.4)	2.5 (1.25–6)	0.07 (0.03–0.29)	54.5 (49.25–58.75)	5 (1.4)/7 (1.9)
Rectal	8 (2.2)	24 (0.7)	2 (1–6)	0.15 (0.03–14.14)	58.5 (47–66.75)	4 (1.1)/4 (1.1)
Esophageal	7 (1.9)	21 (0.6)	1 (1–2)	0.08 (0.04–2.14)	68 (58–71)	6 (1.6)/1 (0.3)
Gastroesophageal	4 (1.1)	27 (0.8)	4 (3.25–13)	0.18 (0.06–1.01)	61.5 (57.75–69.75)	3 (0.8)/1 (0.3)
Gastric	1 (0.3)	2 (0.1)	2	3.22 (2.04–)	66	0/1 (0.3)
Neuroendocrine	1 (0.3)	16 (0.5)	16	0.13 (0.05–0.21)	62	1 (0.3)/0
Duodenal	1 (0.3)	2 (0.1)	2	21.26 (0.28–)	56	1 (0.3)/0
Gallbladder	1 (0.3)	1 (0.0)	1	0.30	59	0/1 (0.3)
Cholangiocellular	1 (0.3)	1 (0.0)	1	8.05	70	0/1 (0.3)
CUP	18 (4.9)	130 (3.9)	1 (1–11.25)	0.13 (0.05–0.68)	62 (56–75.25)	11 (3.0)/7 (1.9)
Sarcoma	9 (2.4)	42 (1.3)	3 (1–6.5)	0.53 (0.16–6.10)	48 (34.5–65.5)	7 (1.9)/2 (0.5)
Liposarcoma	2 (0.5)	8 (0.2)	4 (1–)	0.81 (0.08–20.17)	71.5 (67–)	1 (0.3)/1 (0.3)
Angiosarcoma	1 (0.3)	3 (0.1)	3	8.9 (6.6–)	47	1 (0.3)/0
Ewing sarcoma	1 (0.3)	1 (0.0)	1	5.93	30	1 (0.3)/0
DSRCT	1 (0.3)	19 (0.6)	19	0.46 (0.21–0.89)	39	1 (0.3)/0
Soft tissue sarcoma	1 (0.3)	1 (0.0)	1	13.67	64	1 (0.3)/0
Synovial sarcoma	1 (0.3)	6 (0.2)	6	0.05 (0.02–9.58)	28	1 (0.3)/0
Endometrial stromal sarcoma	1 (0.3)	1 (0.0)	1	36.98	56	0/1 (0.3)
Unknown	1 (0.3)	3 (0.1)	3	12.57 (0.01–)	48	1 (0.3)/0
Head/Neck	2 (0.5)	4 (0.1)	2	6.74 (0.13–35.10)	64 (58−)	1 (0.3)/1 (0.3)
Tonsillar	1 (0.3)	2 (0.1)	2	27.8 (13.3–)	58	1 (0.3)/0
Thyreoid	1 (0.3)	2 (0.1)	2	0.14 (0.12–)	70	0/1 (0.3)

*BM* brain metastasis, *cc* cubic centimeter, *CUP* cancer of unknown primary, *DSRCT* desmoplastic small round cell tumor, *GI* gastrointestinal primary tumor, *GU* genitourinary primary tumor, *N* number of, *NST* invasive carcinoma of no special type

Other systemic metastases than BM were present in 324/369 (87.8%) patients. 42/369 (10.3%) patients developed a carcinomatous meningitis (including 14 (33.3%) patients with pulmonary, 12 (28.6%) with breast, 8 (19.0%) with genitourinary, 4 (9.5%) with skin, 2 (4.8%) with sarcoma, 1 (2.4%) with cancer of unknown primary, and 1 (2.4%) patient with gastrointestinal cancer).

### Frequency and lesion volume of BM

Considering the number of patients with pulmonary and breast cancer in our sample these patients had a disproportionately higher percentage of metastatic lesions compared to patients of other tumor groups: pulmonary cancer accounted for 49% and breast cancer for 20% of all BM, whereas the frequency of pulmonary cancer in the whole collective was 45% and of breast cancer 13%, please refer to Table 1 for further details.

Overall, we detected 3 (1–6.5) BM per patient, with breast cancer patients showing the highest median number of BM per patient (3, 1–11), details are listed in Table 1. In contrast, BM from cancer of unknown primary were mostly solitary. The smallest BM were found in breast cancer and the largest ones were observed in patients with sarcoma and head/neck cancer (Table 1). The median total BM volume load was 5.9 (0.7–16.1) cc per patient.

### BM distribution in the brain

**Fig. 1:**
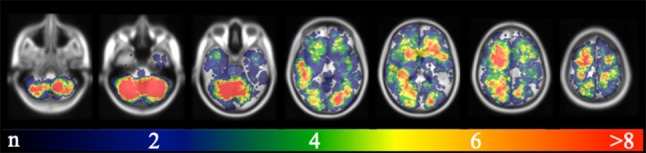
Brain metastases probability map of all 369 patients included in this study of selected brain slices from caudal (left) to cranial (right). The color bar on the bottom indicates the number of BM per area (blue-red colored scale with blue representing one metastasis and red the maximum number of metastases). According to its size, the cerebellum is clearly overrepresented

The majority of BM were located in the frontal lobes (n = 1047/3313; 31.3%), cerebellum (806; 24.6%), parietal (497; 15.0%), temporal (356; 10.7%), and occipital lobes (345; 10.4%). Considering its size, the cerebellum (12.6% of total brain volume) contained a disproportionately high number of BM (Fig. 1).

**Fig. 2:**
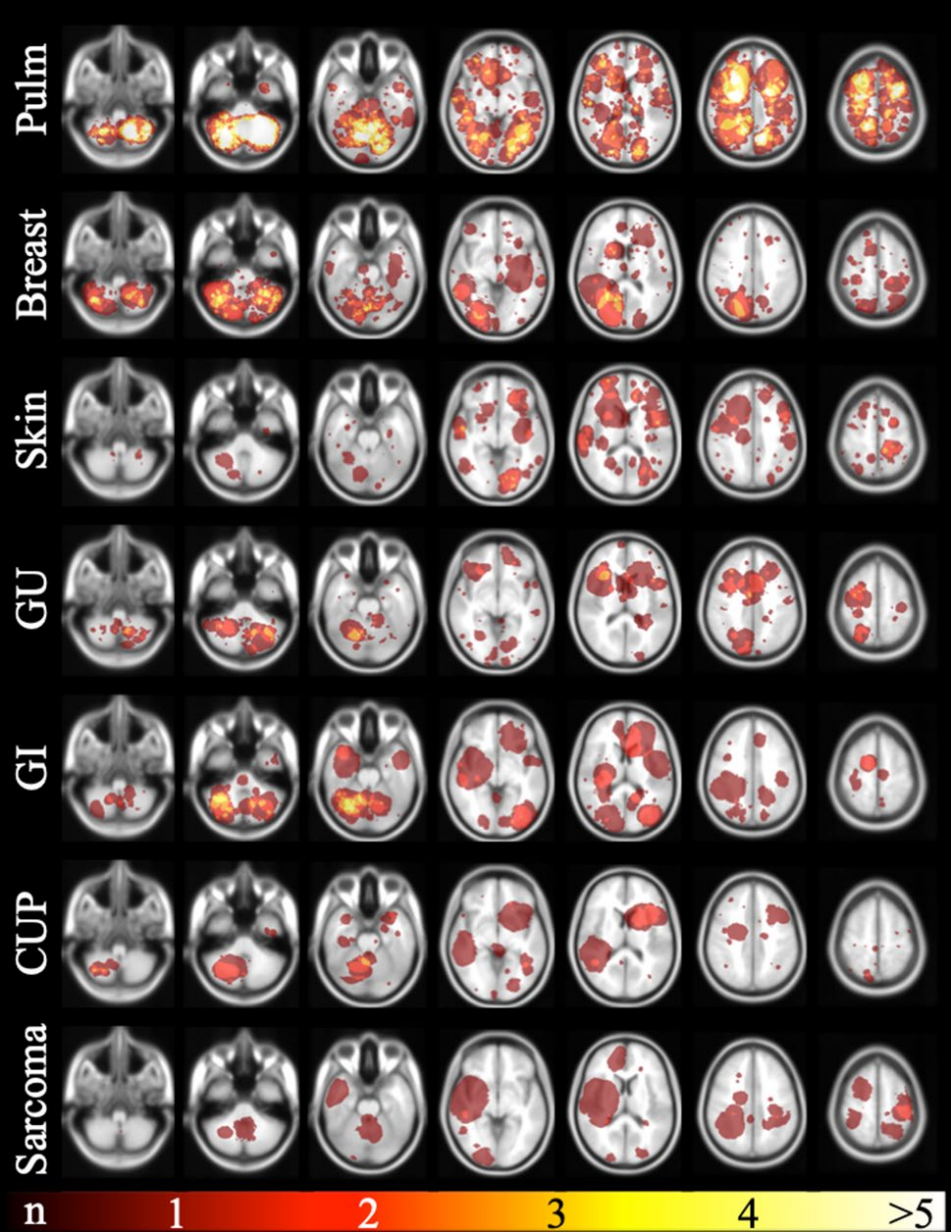
Brain probability maps of the different primary tumors. The selected brain slices from caudal (left) to cranial (right) are identical to Fig. 1. Each row represents another primary tumor group. The top row depicts brain metastases distribution of the pulmonary cancer patients representing the largest primary tumor group. The red-yellow colored scale on the very bottom indicates the number of BM per area (with red representing one metastasis and yellow the maximum number of metastases per area). The primary tumor group “head and neck “ is not shown. *Pulm* pulmonary primary tumor, *GU* genitourinary primary tumor, *GI* gastrointestinal primary tumor, *CUP* cancer of unknown primary

BM distribution varied based on the primary tumor group: BM from pulmonary and gastrointestinal cancer favored the infratentorial area whereas BM from skin cancer and sarcoma were preferably located in the supratentorial space. Breast BM showed a high affinity to structures supplied by the posterior circulation areas. For further details of regional distribution by primary tumor type please refer to Fig. 2. Due to very small patient numbers in the tumor group “head and neck” we waived metastases mapping here.

### Multivariate analysis

Mixed effects logistic regression model analysis revealed a significant impact of certain primary tumor types on lesion location whereas age and sex had no influence: BM from skin cancer accumulated in the frontal lobes (OR 1.518, probability 42.9%, CI 34.5–51.8%, p = 0.037) and clearly avoided the cerebellum (OR 0.215, probability 4.6%, CI 2.3–8.7%, p < 0.001).

BM from both breast and gastrointestinal cancer showed an opposite distribution to skin cancer BM: they favored the cerebellum (breast BM: OR 2.161, probability 32.4%, CI 23.3–43.0%, p = 0.006; gastrointestinal BM: OR 2.117, probability 31.9%, CI 21.3–44.9%, p = 0.016) and were rarely found in the frontal lobes (breast BM: OR 0.487, probability 19.4%, CI 14.5–25.6, p < 0.001; gastrointestinal cancer: OR 0.572, probability 22.1%, CI 15.2–31.0%, p = 0.025).

**Fig. 3:**
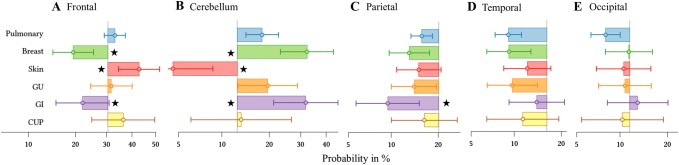
Horizontal boxplots showing the probability of occurrence of metastases for a pulmonary, breast, skin, genitourinary, gastrointestinal, and unknown primary (from top to bottom) for each of the five largest locations (**a** frontal; **b** cerebellum; **c** parietal; **d** temporal; **e** occipital). The vertical line in **a**–**e** represents the percentage of the respective lobe volume of the whole brain volume in the MNI space (or: the brain metastases probability that would statistically be expected in this region) with boxes to the right demonstrating a higher and boxes to the left a lower probability. The stars indicate statistical significance. The primary tumor groups “sarcoma” and “head and neck “ are not shown. *GU* genitourinary primary tumor, *GI* gastrointestinal primary tumor, *CUP* cancer of unknown primary

Furthermore, gastrointestinal BM were extremely infrequent in the parietal lobes (OR 0.555, probability 9.4%, CI 5.7–15.3%, p = 0.045), Fig. 3.

## Discussion

We aimed to evaluate BM distribution originating from various tumor entities. In a cohort of 369 patients we were able to demonstrate that the primary tumor type is highly relevant in the spatial distribution of BM. Most strikingly, BM from skin cancer showed an almost exclusive affinity to the supratentorial space confirming our initial hypothesis.

The spatial arrangement patterns of BM described here represent the final stage of BM formation. The steps required for BM formation are not yet fully understood. Briefly summarized, these include: invasion of the cancer cell into the surrounding tissue, intravasation and systemic spread, metastatic cell arrest at vascular branches/adhesion, extravasation, and angiogenesis [24, 25]. On the one hand, molecular and genetic features of the tumor cell determine if metastatic progression is successful or not; on the other hand there are systemic and brain microenvironmental requirements to complete BM formation (“seed and soil hypothesis”) [26]. Previous studies showed that the primary tumor type influences both “seed” and “soil” of BM development [24, 25]. As an example for the “seed” aspect, melanoma cells were shown to recruit preexisting vessels in the brain parenchyma (vessel-cooption) whereas pulmonary cancer cells induce neoangiogenesis mediated by vascular endothelial growth factor A [24]. Two imaging studies demonstrated an inverse correlation between the occurrence of cerebral microangiopathy (“soil”) and BM of multiple tumor entities [27, 28].

Aforementioned arterial hematogenous spread is the main route for metastatic disease in the brain [5]. Less common ways are by direct growth from head and neck malignancies and perineural spread along cranial nerves that is predominantly found in skin cancer [29, 30]. We think that both forms are not particularly relevant for our patient collective since the BM from the two head and neck primary tumors were distant from each other and perineural tumor dissemination primarily results in neural thickening and enhancement rather than solid metastases [29, 30]. It was only recently shown that the brain possesses a lymphatic system draining into cervical lymph nodes [31]. To the best of our knowledge, there is no evidence of afferent lymphatic vessels carrying fluids to the brain, thus making lymphatic dissemination of cancer cells into the brain unlikely.

There are some drawbacks of our study. First, we summarized tumors of different biological origin into large primary tumor groups in order to create a clearly structured visual overview. In our opinion this simplification was necessary especially for tumors leading only rarely to BM to facilitate a practical visual representation of BM distribution. Furthermore, due to the single-center nature of the study, we could include only limited numbers of patients with less frequent tumor entities. Second, we did not perform histological or molecular subgroup analysis that would eventually be needed for clinical application. Interestingly, Takano et al. were able to demonstrate that distribution of pulmonary cancer BM depend on mutation status of epidermal growth factor receptor [20]. This suggests a major influence of molecular biological determinants of the cancer cell. Since this is the first work analyzing major solid primary tumor groups we aimed to create a “general” atlas as a primary step. In order to sub-stratify tumor types our database needs to be expanded. Third, we included patients irrespective of their disease stage. Thus, we could not determine whether the first BM of a patient differs in location predilection from later BM.

Besides identifying the possible primary tumor entity during diagnostics knowledge of BM distribution could improve radiation therapy by shrinking the irradiation field to the tumor-specific BM distribution areas instead of WBRT. This is even more important since it is estimated that incidence of BM will continue to increase in line with advanced systemic treatment strategies leading to prolonged life expectancy of cancer patients [32].

With our study, we were able to demonstrate a significant impact of primary tumor entity on the spatial distribution of brain metastases. Our results underline the importance of further research elucidating the mechanism underlying metastases formation. Understanding the molecular basis of the patterns observed here will be key to a future treatment approach.

## Electronic supplementary material

Below is the link to the electronic supplementary material.

Electronic supplementary material 1 (XLS 334 kb)
